# Clinical and Biological Markers in Hypereosinophilic Syndromes

**DOI:** 10.3389/fmed.2017.00240

**Published:** 2017-12-22

**Authors:** Paneez Khoury, Michelle Makiya, Amy D. Klion

**Affiliations:** ^1^Human Eosinophil Section, Laboratory of Parasitic Diseases, National Institute of Allergy and Infectious Diseases, National Institutes of Health, Bethesda, MD, United States

**Keywords:** eosinophil, biomarkers, hypereosinophilic syndrome, eosinophilic esophagitis, eosinophilic granulomatosis with polyangiitis, eosinophilia, eosinophilic disorders

## Abstract

Hypereosinophilic syndromes (HES) are rare, heterogeneous syndromes characterized by markedly elevated eosinophil counts in the blood and/or tissue and evidence of eosinophil-associated pathology. Although parasitic infections, drug hypersensitivity, and other disorders of defined etiology can present as HES (associated HES), treatment is directed at the underlying cause rather than the eosinophilia itself. A number of additional subtypes of HES have been described, based on clinical and laboratory features. These include (1) myeloid HES—a primary disorder of the myeloid lineage, (2) lymphocytic variant HES—eosinophilia driven by aberrant or clonal lymphocytes secreting eosinophil-promoting cytokines, (3) overlap HES—eosinophilia restricted to a single organ or organ system, (4) familial eosinophilia—a rare inherited form of HES, and (5) idiopathic HES. Since clinical manifestations, response to therapy, and prognosis all differ between HES subtypes, this review will focus on clinical and biological markers that serve as markers of disease activity in HES (excluding associated HES), including those that are likely to be useful only in specific clinical subtypes.

## Introduction

Hypereosinophilic syndromes (HES) are defined by the presence of hypereosinophilia [absolute eosinophil count (AEC) > 1,500/μL or marked tissue eosinophilia] and eosinophil-associated clinical manifestations. Various clinical subtypes of HES have been described based on the etiology of the eosinophilia (primary, secondary, or unknown) and clinical features (systemic or organ-restricted) (1). Although HES can occur in the context of defined disorders, such as drug hypersensitivity, helminth infection, and neoplasia, for which specific treatment of the underlying secondary cause leads to resolution of the eosinophilia (associated HES), for the purposes of this review, HES refers to all clinical subtypes of HES with the exception of associated HES.

The development of standardized clinical assessments of disease activity, such as patient-reported outcomes (PROs) and clinician-reported outcomes (ClinROs), that can be used to guide treatment and serve as clinical trial endpoints has been complicated in HES due to the heterogeneity of disease across HES subtypes and organ systems and the rarity of the disorder itself. Although significant progress has been made in the development of these tools in organ-restricted eosinophilic disorders, such as eosinophilic esophagitis (EoE) (2–5), these subtype-specific PROs and ClinROs are not broadly applicable to the overall HES population. This has, in turn, hampered the development of surrogate markers of disease activity in HES.

Given the central role of eosinophils in HES, quantification of eosinophil numbers in the blood or tissue would seem the most logical method to monitor disease activity in HES and is, in fact, the most common biomarker used in clinical practice. Despite this, AEC has not been widely accepted as a surrogate of disease activity in clinical trials of HES, particularly those involving novel therapies that specifically target eosinophils but may or may not affect clinical outcomes. Clearly, additional biological markers are needed. This review is divided into two parts. The first section will focus on data pertaining to biomarkers related to eosinophilia and eosinophil activation as general indicators of disease activity in HES. This will be followed by a discussion of biomarkers relevant to selected subtypes of HES, but unlikely to be generalizable to HES as a whole.

## General Biomarkers of Disease Activity in HES

Eosinophils are characterized by the presence of eosin-avid secondary granules containing cationic granule proteins [major basic protein (MBP), eosinophil cationic protein (ECP), eosinophil peroxidase (EPO), and eosinophil-derived neurotoxin (EDN)] and a wide array of cytokines and chemokines. When released into the tissues by activated eosinophils, these mediators, together with reactive oxygen species and lipid mediators, can lead to tissue damage and the end organ manifestations of HES. As mentioned earlier, a major controversy in clinical trial endpoint design in HES has been whether reduction in AEC is an appropriate surrogate marker of disease activity. The fact that some individuals with hypereosinophilia (AEC > 1,500/μL) are asymptomatic and do not develop end organ manifestations (6) has been cited as evidence that biomarkers of eosinophil activation or tissue infiltration might be more useful in this regard. Available data addressing this question are summarized below.

### Absolute Eosinophil Count

The association between elevated peripheral eosinophil counts and clinical pathology was first noted at the turn of the century by Loeffler who described a characteristic form of endomyocardial fibrosis in association with blood eosinophilia (7). Subsequent case series, using persistent AEC > 1,500/μL as a defining criterion for HES, noted an association between extremely elevated AEC (white blood cell counts >100,000/μL) and poor prognosis (8, 9). Consistent with these findings, patients with *PDGFRA*-positive myeloid neoplasm, one of the most aggressive forms of HES, have higher AECs than patients with other clinical subtypes of HES and dramatic resolution of clinical manifestations following normalization of the AEC with imatinib therapy (10).

Despite these findings and the large body of circumstantial evidence from case reports, case series and clinical practice documenting an association between the resolution of clinical manifestations of HES and normalization of the AEC, assessment of the AEC as a surrogate marker of disease activity has not been studied directly in the context of clinical trials to date. That said, the efficacy of mepolizumab as a steroid-sparing agent was associated with reduction of AEC in two placebo-controlled, double-blind trials in HES [one in subjects with steroid-responsive HES (11) and the second in subjects with eosinophilic granulomatosis with polyangiitis (EGPA) (12)], suggesting that AEC is a useful marker of disease activity. The results of ongoing and recently completed trials (NCT02130882; NCT02101138) in HES using agents that selectively target eosinophils should provide additional support for the utility of the AEC as a biomarker of response.

### Tissue Eosinophilia

Although tissue eosinophilia would seem to be a more specific indicator of disease activity in HES, the utility of eosinophil quantification in tissue biopsies to monitor disease activity is hampered by the difficulty in obtaining samples, the patchy nature of eosinophilic tissue infiltration, and the fact that intact eosinophils may be absent despite clear evidence of their involvement by immunohistochemical staining for eosinophil granule proteins (EGPs) (13–16). To date, the best data associating tissue eosinophil numbers with clinical symptomatology come from EoE where the numbers of eosinophils in normal tissue have been defined (17), and suppression of tissue eosinophil counts has been associated with improved long-term prognosis (18). Unfortunately, despite encouraging data from a small open-label trial (19), randomized placebo-controlled trials using anti-IL-5 antibody therapy (mepolizumab and reslizumab) have not demonstrated an association between reduction in tissue eosinophilia and improved symptoms (20–22). Potential explanations for the lack of symptomatic improvement include incomplete depletion of tissue eosinophilia, involvement of other cell types and/or structural changes due to fibrosis and remodeling that may require a longer time frame for resolution.

### Eosinophil Granule Proteins

Released during eosinophil activation and deposited in tissue in sites of eosinophilic inflammation, EGPs are attractive candidate biomarkers for the monitoring of disease activity in HES (23). They can be detected and quantified in the blood (24–26), body fluids (27), and tissue (13–15, 28–31) using various immunoassays, and blood and/or body fluid levels have been shown to correlate with tissue deposition of EGP in a wide range of HES, including EoE in the absence of peripheral eosinophilia (24).

There are a several biologically relevant differences between EDN, EPO, MBP, and ECP. MBP is the predominant protein in the core of the eosinophil secondary granule. It exists as two highly basic homologs, MBP-1 and MBP-2 (26), both of which circulate as neutral pH pro-proteins. Of note, most immunoassays do not distinguish between pro-MBP and MBP. Whereas EPO is quite specific for the eosinophil lineage, MBP-1, EDN, and ECP are also present in neutrophils and/or basophils albeit at lower levels (32, 33). This does not appear to affect their ability to be used as a proxy for eosinophil-associated tissue pathology in most settings but deserves mention.

Immunohistochemical staining of tissue for EGP has been extremely useful in clarifying the role of eosinophils in the pathogenesis of HES when intact eosinophils are not detectable. Moreover, EGP staining has been shown to correlate with disease activity in some settings. For example, in one study, serial skin biopsies from patients with episodic angioedema with eosinophilia demonstrated EGP staining only when symptoms were present (15, 34). Unfortunately, the utility of EGP tissue staining as a biomarker of disease activity is limited by the need for serial tissue sampling. To address this issue in EoE, a number of novel and less invasive techniques have been developed. These include the esophageal string test (35) and the cytosponge (36). Both techniques involve swallowing a string (in the case of the cytosponge, this is attached to a gelatin capsule containing a mesh) from which EGP can be eluted and quantified. Good correlation between eluted EGP levels and immunohistochemical staining of matched biopsies has been confirmed for both techniques (37). Finally, ultrasound visualization of granule protein density using MBP-1 labeled-insulin particles has been demonstrated in *ex vivo* monkey esophagi and may ultimately provide a third non-invasive tool for the measurement of tissue EGP in EoE (38). The applicability of these or similar techniques to other tissues remains to be seen.

The utility of measuring circulating levels of EGPs to monitor disease activity in HES has been somewhat controversial in large part due to the lack of standardization of sample collection (eosinophil lysis could lead to falsely elevated levels) and differing assay parameters between studies. Nevertheless, there are some data to suggest that circulating EGP levels have value in the monitoring of disease activity in HES. An interesting observation in this regard has been the association of elevated EDN and EPO levels with clinical manifestations rather than peak eosinophil count in patients with episodic angioedema and eosinophilia (24). A similar association between clinical disease and elevated EDN levels was seen in a study comparing subjects with asymptomatic familial eosinophilia to subjects with active HES (39).

Data from clinical treatment trials have been more difficult to interpret. Whereas decreases in serum EDN levels were reported in subjects who received active drug in a placebo-controlled trial of mepolizumab in HES (11) and ECP levels decreased in response to mepolizumab therapy in three patients with HES and eosinophilic dermatitis (40), AEC also decreased in both studies making it difficult to assess the added benefit of measuring EGP levels. Moreover, in a recent study of non-invasive biomarkers of EoE, AEC, but not serum levels of ECP, was predictive of residual disease following topical steroid therapy (41).

Measurement of EGP in body fluids, such as urine, that do not normally contain eosinophils, has the theoretical advantage of eliminating false positive results due to eosinophil lysis. Although there are no studies examining urine levels of EGP in HES to date, several small studies in atopic dermatitis (42, 43) demonstrated a correlation between urine levels of ECP and clinical disease severity. By contrast, no such relationship was noted in children with asthma (44).

### Eosinophil Surface Receptors

A wide variety of eosinophil surface markers are reported to be up- or downregulated on activated eosinophils (23, 45), Many of these, including IL-5Rα, CD69, and CD44, have been shown to have altered expression on eosinophils from patients with HES, but also in patients with HE_US_ (39). Despite this, there are little longitudinal data assessing changes in expression of these activation markers in response to therapy in patients with HES. In a single study in EoE, expression of activation markers on blood eosinophils was unchanged by topical steroid therapy, although the effect of therapy on esophageal eosinophilia was not reported (46).

### Serum Cytokines, Chemokines, and Soluble Receptors

**Figure 1:**
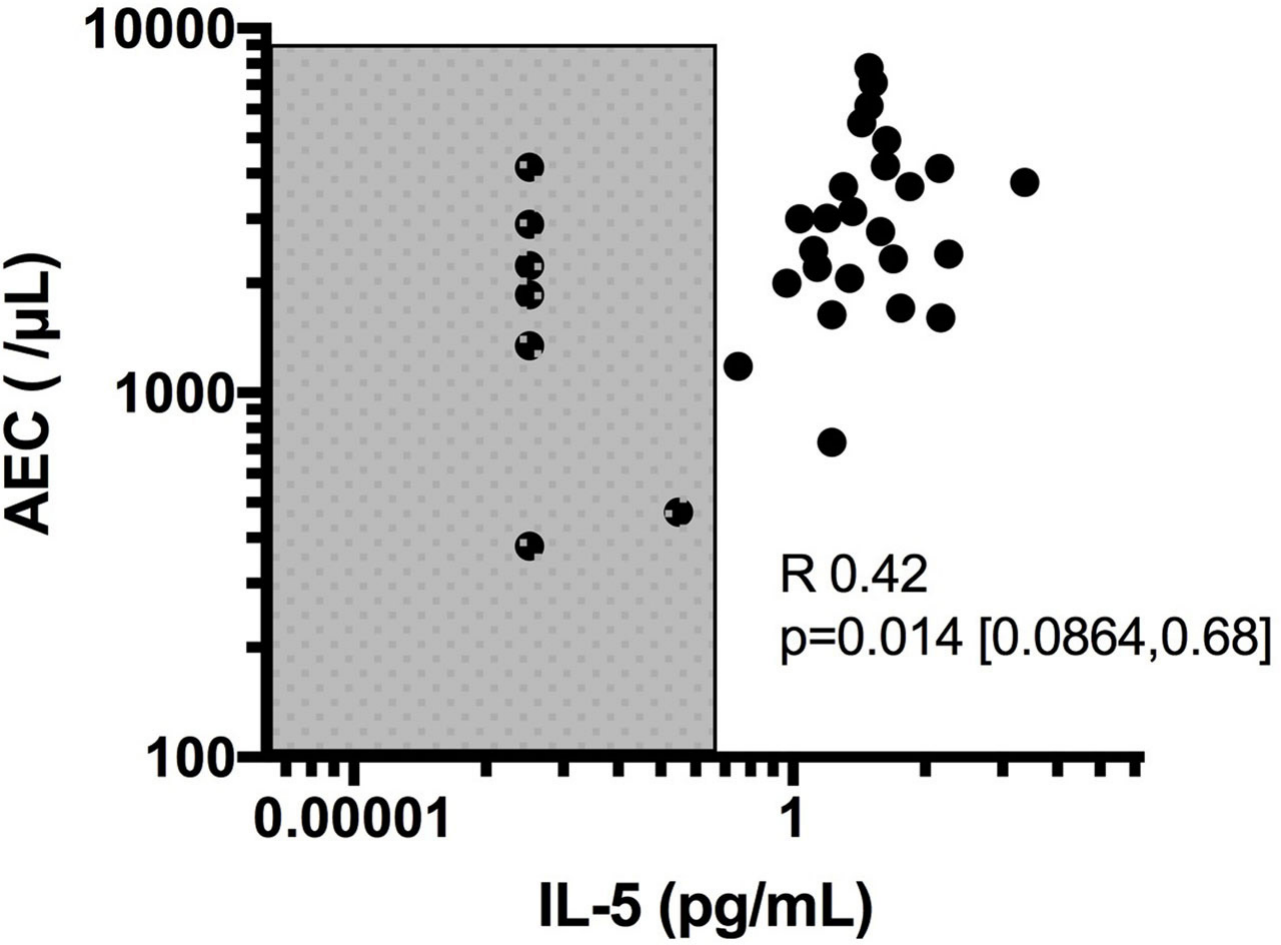
Correlation of absolute eosinophil count (AEC) with IL-5 levels and demonstration of subjects with undetectable IL-5 despite elevated eosinophil counts. Shaded area denotes values below the limit of detection of IL-5 (0.1 pg/mL) in the assay used.

Despite its clear role in the production, activation and regulation of eosinophils, IL-5 has been disappointing as a biomarker of disease activity in HES. Although IL-5 levels correlate with AEC overall, serum IL-5 is undetectable in some patients with untreated HES [Figure 1, unpublished data from Ref. (47)]. In this regard, serum IL-5 levels do not contribute additional information when the AEC is known. In addition, increased serum IL-5 levels have been reported in the setting of clinical and hematologic remission following administration of several different biologics designed to target eosinophils, including mepolizumab and benralizumab (48, 49). The reasons for this are likely multifactorial and include measurement of IL-5/anti-IL-5 immune complexes (mepolizumab) and antibody blocking of IL-5 binding to its receptor (benralizumab). Soluble IL-5R is measurable in the serum of most, if not all, patients with HES, and levels are correlated with serum IL-5 levels (47). Whether this would provide a better biomarker of disease activity, owing to its reliable detection in serum in contrast to IL-5, remains to be seen. Finally, a number of studies have looked at other serum cytokines and chemokines as biomarkers of disease activity in HES. Of these, mediators of potential interest have been identified mostly in EGPA and include IL-25 (50), serum CCL17/thymus and activation-regulated chemokine (TARC) levels (51, 52), and CCL26/eotaxin-3 (53, 54). Interestingly, despite tissue data implicating CCL26/eotaxin-3 in the pathogenesis of EoE, serum levels of these mediators were not increased in EoE patients and were not altered by therapy (55). Finally, although some authors have reported elevated IL-3 in the plasma of patients with eosinophilia in conjunction with intracellular staining in CD8+ T cells (56), IL-3 is not universally detected in serum (57) of patients with HES, and the role of IL-3 as a biomarker in HES remains to be explored.

### Omics

An exciting advance in biomarker development has been the use of molecular profiling techniques to identify patterns of expression that can be used to follow disease activity. This approach has been used successfully in EoE, where patterns of gene expression in esophageal biopsies have led to the development of an EoE molecular diagnostic panel (EDP) (58, 59) which is further discussed in the accompanying review, and a microRNA signature (60) that correlate with disease activity and response to therapy.

## Clinical Subtype-Specific Biomarkers in HES

Over the past decade, it has become increasingly apparent that there are distinct clinical subtypes of HES that differ in their etiologies, clinical manifestations, and responses to therapy. These are more extensively discussed in the companion article by Lefevre (61). Whereas the biomarkers discussed herein are relevant to eosinophilic disorders and HES in general, additional markers have been described that have utility restricted to a particular HES clinical subtype.

### Lymphocytic Variant HES (LHES)

**Figure 2:**
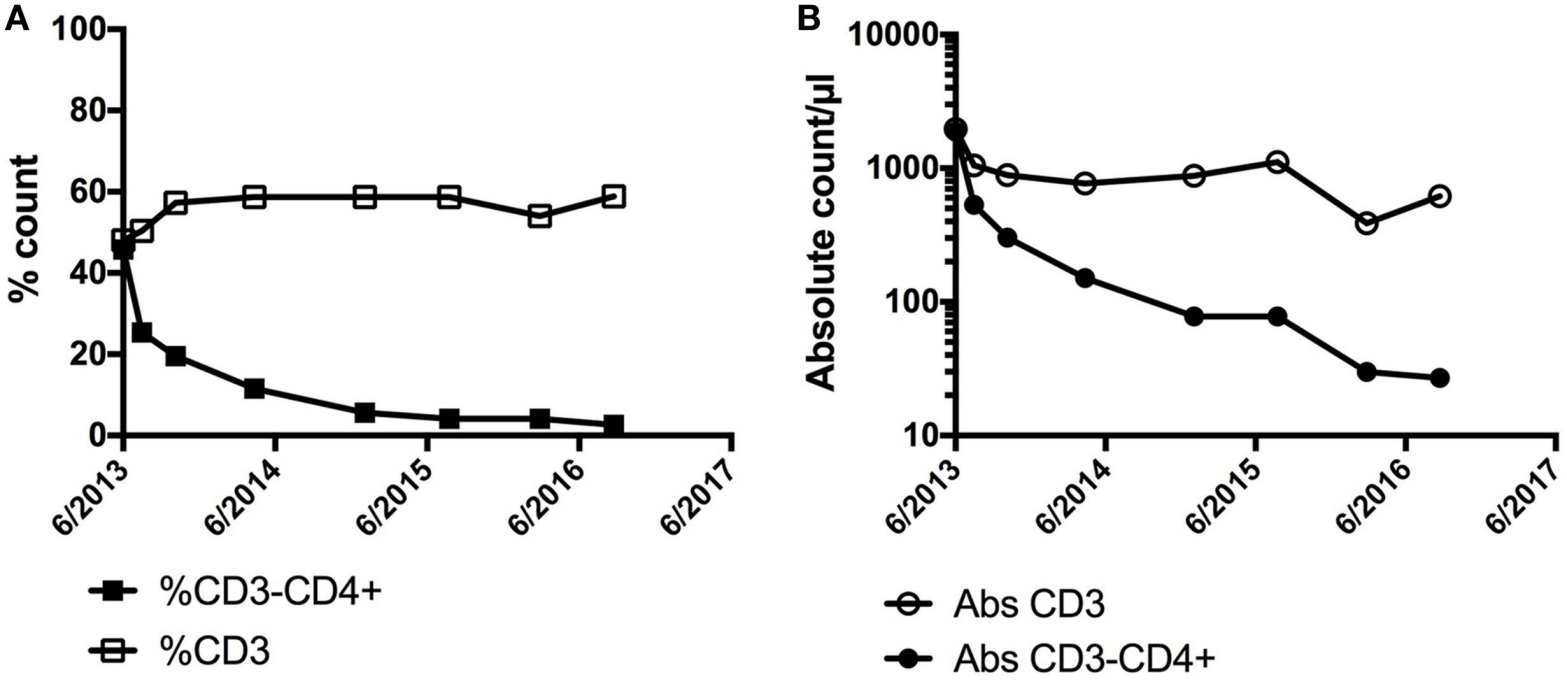
Demonstration of a preferential decline in the **(A)** % of CD3−CD4+ and **(B)** absolute count of CD3−CD4+ cells in a patient with LHES after initiation of interferon-alpha with associated clinical improvement in skin involvement. No significant change was noted in the % or absolute counts of CD3+ cells overall.

LHES is defined by the presence of a clonal or aberrant phenotypic T cell that secretes type 2 cytokines driving the eosinophilia and elevated serum IgE levels seen in this clinical variant (62, 63). Whereas the most common aberrant immunophenotype is CD3−CD4+, various aberrant immunophenotypes have been described (64), and some patients have cytokine-secreting clonal T-cell populations despite an apparently normal immunophenotype. Dermatologic manifestations, including angioedema, nodules, eczematous dermatitis, and erythroderma, are common in patients with LHES (65), and aberrant T cells can often be detected in skin biopsies from affected areas (66). Patients with LHES are often glucocorticoid responsive but typically require moderately high doses (67). Consequently, glucocorticoid-sparing agents with effects on T cells, such as interferon-alpha and cyclosporine, are frequently used. Although LHES is considered a benign lymphoproliferative disorder, a small proportion of LHES patients eventually develop a lymphoid malignancy, often heralded by expansion of the aberrant clonal T-cell population (62, 68). Conversely, regression of the aberrant T-cell population can be seen in response to effective therapy (Figure 2). Elevations in serum CCL17/TARC are more frequent in patients with LHES (69, 70), but information is lacking on the use of CCL17/TARC levels to monitor disease activity.

### Myeloid HES (MHES)

Myeloid HES refers to the subgroup of patients with HES in the setting of a primary myeloid disorder. Although most of these patients have detectable molecular abnormalities (most commonly the fusion gene *FIP1L1-PDGFRA*), others have a similar clinical phenotype of unknown cause. Clinical and laboratory features associated with MHES include dysplastic eosinophils, anemia and/or thrombocytopenia, elevated serum tryptase and B12 levels, and bone marrow features suggestive of a myeloid neoplasm (10). Before the availability of imatinib (a tyrosine kinase inhibitor with activity against *PDGFRA* and *PDGFRB*), mortality rates in patients with MHES were extremely high, due primarily to endomyocardial fibrosis and thromboembolic events. Currently, remission rates on imatinib therapy approach 100% in patients with *PDGFR*-associated disease and up to 50% in patients with other forms of MHES.

Although the AEC normalizes with effective therapy in MHES and can be used to monitor disease activity, data from chronic myelogenous leukemia and drug interruption trials in *PDGFRA-*associated HES suggest that molecular monitoring is preferable when possible since molecular relapse may precede hematologic (and clinical) relapse by several months (71). This is particularly important in view of recent data demonstrating sustained remission after imatinib discontinuation in some patients (72–74).

### Overlap HES

Overlap HES includes single organ and/or defined disorders that are characterized by eosinophilia and eosinophil-associated pathogenesis, including eosinophilic gastrointestinal disorders and EGPA. These disorders have distinct clinical presentations and complications and, for this reason, are often approached differently than other forms of HES. Although potential biomarkers for these conditions include the previously discussed general markers of eosinophilia and eosinophil activation, additional disease-specific issues are discussed below.

#### Eosinophilic Esophagitis

Despite significant advances in the development of biomarkers for EoE, the lack of correlation between the number of eosinophils in tissue and clinical symptoms remains a problem, particularly with regard to clinical trial design. Consequently, there has been increasing interest in the development of additional objective measures to assess improvement of long-term sequelae. One such tool is EndoFLIP^®^ (endolumenal functional lumen imaging probe), an inflatable balloon that measures the cross-sectional area and intraluminal pressure of the esophagus while under distension (as if a solid bolus was present). Using this technique, reduced distensibility was demonstrated in patients with dysphagia or a history of impaction as compared with healthy controls (75). Interestingly, decreased distensibility did not correlate with mucosal eosinophilia (75) but did correlate with ring severity and impactions (76).

#### Eosinophilic Granulomatosis with Polyangiitis

Several studies have examined the use of standard laboratory markers of inflammation, including erythrocyte sedimentation rate and C-reactive protein, in EGPA. Although these markers have been shown to be elevated in active disease at the population level, a longitudinal study using a validated ClinRO as the gold standard found that they were affected by disease severity and treatment status, limiting their success in predicting disease activity and relapse at the individual patient level (77).

## Conclusion

With the advent of targeted therapies that reduce blood eosinophilia but may have varied effects on tissue eosinophilia and eosinophil-related end organ manifestations, there is an increasing need for reliable, non-invasive markers of disease activity in HES. Although some progress has been made in select subtypes of HES, including EoE and *PDGFRA*-positive myeloid neoplasm, generally applicable, validated biomarkers in HES are lacking. This is likely due, at least in part, to the heterogeneity of clinical manifestations, lack of understanding of the factors driving the varied HES subtypes and paucity of longitudinal studies addressing this issue. Although not validated as a surrogate marker for disease activity in HES, the AEC remains a key laboratory test that is used by experts to assess disease activity and response to therapy in all HES subtypes. Soluble mediators that correlate with active disease, including serum levels of EGP, have been identified, although increased predictive value compared to the AEC has not been demonstrated in most cases and none have been validated in prospective double-blind clinical trials to date. Finally, tissue-based markers, including tissue eosinophilia, granule protein deposition, and transcriptome analysis, have demonstrated utility in monitoring disease activity in some settings but are limited by the availability of appropriate tissue samples. While the development of novel non-invasive sampling methods and global approaches to biomarker discovery (“omics”) are exciting, carefully designed clinical trials are clearly needed to validate existing and novel biomarkers for accurate monitoring and assessment of therapeutic interventions.

## Ethics Statement

The data presented in this manuscript were collected under research protocol NCT00001406. This study was carried out in accordance with the recommendations of the Belmont Report with written informed consent from all subjects. All subjects gave written informed consent in accordance with the Declaration of Helsinki. The protocol was approved by the NIAID Institutional Review Board.

## Author Contributions

AK, PK, and MM each contributed to the writing of the manuscript.

## Conflict of Interest Statement

The authors declare that the research was conducted in the absence of any commercial or financial relationships that could be construed as a potential conflict of interest. The reviewer PA declared a past collaboration with the authors to the handling Editor.
